# Modeling Time to Blindness of Glaucoma Patients: A Case Study at Jimma University Medical Center

**DOI:** 10.34172/jrhs.2022.83

**Published:** 2022-05-11

**Authors:** Meskerem Getachew Gebremariam, Reta Habtamu Bacha, Demeke Kifle Demissie, Kibrealem Sisay Wolde, Kenenisa Tadesse Dame, Geremew Muleta Akessa

**Affiliations:** ^1^Department of Statistics, College of Natural Sciences, Jimma University, Jimma, Ethiopia

**Keywords:** Archimedean copula families, Ethiopia, Glaucoma, Kendall’s tau, Time to blindness

## Abstract

**Background:** Glaucoma is a significant public health problem due to its substantial increase in the projected number of glaucoma cases. In Ethiopia, glaucoma accounts for 5.2% of irreversible blindness and is the fifth main cause of blindness. The main objective of this study was to modeling time to blindness of left and right eyes of glaucoma patients.

**Study Design:** An institution-based retrospective cohort study.

**Methods:** This study was conducted among 315 glaucoma patients admitted to the Ophthalmology Department of Jimma University Medical Center (JUMC), Southwest Ethiopia, from January 1, 2016, to August 30, 2020. Kaplan-Meier survival analysis and semiparametric and parametric copula models were applied to identify factors that affect time to the blindness in glaucoma patients and the dependence between time to the blindness of the left and right eyes, respectively. An Akaike information criterion (AIC) was used to select the best non-nested model.

**Results:** In total, 211 (66.9%) out of 315 glaucoma patients were blind, whereas 104 (33.1%) patients were censored. The median time to the blindness of the left and right eyes was determined to be 12 months. The result suggested that the risk of the blindness in male patients was 1.005 (*P*=0.01) times higher than that in female patients, and the risk of the blindness in patients who had early, moderate, and advanced glaucoma was estimated to be 0.582 (*P*=0.002), 0.485 (*P*=0.001) and 0.887 (*P*=0.003) times less than that in the patients with absolute glaucoma, respectively.

**Conclusions:** Age, place of residence, gender, type of medication, diabetes disease, stage of glaucoma, duration of treatment, intraocular pressure (IOP), and cup-disk ratio were significantly associated with and affected by the time to the blindness of left and right eyes in glaucoma patients. Awareness should be given to the community to reduce the burden of glaucoma.

## Background

 Glaucoma is one of the most common causes of blindness and patterns of visual field loss due to retinal ganglion cell degeneration.^1^ Risk factors for the two main types of glaucoma, (i.e., open-angle and closed-angle glaucoma disease) with different patterns of disease occurrence include increasing age, high intraocular pressure (IOP), family history of glaucoma, race and ethnicity, diabetes, as well as female gender.^2-13^Glaucoma imposes a substantial burden on society in terms of higher medical costs, lost productivity, patient morbidity, and the number of ophthalmic consultations^14^and results in different psychological problems, including depression, inferiority complex, anxiety, and repudiation due to the feeling of low self-esteem.^15^

 Glaucoma is a major public health problem that accounts for 8% of the world’s irreversible blindness and is the second largest cause of blindness following cataracts.^16^ An estimated number of people with glaucoma is 60.5 million worldwide, about 13.9% of whom had become blind due to glaucoma.^16^ Moreover, the number of people with glaucoma is expected to escalate to 111.8 million by 2040.^1,17^ In Africa, glaucoma is responsible for 15% of the world’s blindness.^1^ In Ethiopia, an estimated 62 thousand people suffer from irreversible sight loss caused by glaucoma, as the fifth most common cause of blindness.^18,19^

 There is a dearth of published studies on glaucoma in Ethiopia, particularly in the study area with problems in routine health services. Therefore, this study aimed to provide a modeling of time to blindness of the left and right eyes of glaucoma patients considering the different factors that affect the time to the blindness in glaucoma patients. The time to the blindness of the left and right eyes in glaucoma patients can be predicted and statistically estimated using the bivariate survival model.^20^ In this study, various techniques were considered for bivariate survival analysis, as a statistical tool for the analysis of time to the blindness of glaucoma patients’ data. Bivariate survival data is a term used to describe the data that measure the time to a given event of interest. In this study, the event of interest was the time of the blindness of the left and right eyes of glaucoma patients.

 This study aimed to determine the dependence between the time of the blindness of the left and right eyes using the Copula model popularized by Clayton.^21^This model is an important tool for bivariate survival data and estimates the dependence between variables. In this study, the copula model has been used to couple the marginal survival functions of two cluster observations and form a joint survival function. Parametric Archimedean copula models were also considered to estimate the dependence between the time of the blindness of left and right eyes of glaucoma patients.

 The advantage of the parametric method over the semi-parametric method is that, having a baseline distribution for parametric method was good for the sake of simplicity and completeness of further statistical analysis like, quantiles and so on, which account for the popularity of parametric distributions.^22,23^

## Methods

###  Study area, design, and period

 This study has been conducted at the Ophthalmology Department of Jimma University Medical Center (JUMC), which is one of the oldest hospitals in Ethiopia and the only teaching and referral hospital in the southwestern part of the country. Currently, JUMC with an 800-bed capacity provides many health care services for at least 15 million people in its catchment area. This institution-based retrospective cohort study was performed from January 1, 2016, to August 30, 2020.

###  Data collection 

 The data card (related to both left and right eyes of the glaucoma patients) extracted from the ophthalmic patients included such information as socio-demographic and clinical information collected from January 1, 2016, to August 30, 2020, at JUMC, Southwest Ethiopia.The starting point was when the patients started follow-up or were diagnosed at the hospital, and the occurrence of blindness was the endpoint. Admission records were obtained from a total of 315 glaucoma patients.

###  Ethical clearance

 The study protocol was approved by the Research Ethics Review Board of Jimma University, College of Natural Sciences, and the medical director of the Hospital. Due to the retrospective nature of the study, the requirement for obtaining written informed consent from the study participants was waived by the Ethics Review Board, and data were kept anonymous and confidential.

###  Study population and variables

 The study population included all glaucoma patients registered at the Ophthalmology Department of JUMC, Southwest Ethiopia, with regular follow-ups from January 1, 2016, to August 30, 2020. The response variable of the study was the time of the blindness of left and right eyes of glaucoma patients, measured in months between the patient’s admission to the hospital and the occurrence of left and right eye blindness. Right censoring was applied in this study. The exclusion criteria included patients who lost to follow-up during the study period, those who withdrew from the study, and the subjects who did not experience the event before the termination of the study. The events in this study included the occurrence of blindness in the left and right eyes. The independent variables included age, gender (female, male), place of residence (rural, urban), diabetes disease (no, yes), type of medication (Timoglue, Diamox, and Timolol), duration of treatment (short, medium, and long), stage of glaucoma (early, moderate, advanced, and absolute), cup-disc ratio (≤ 0.7, > 0.7), and IOP (normal and not normal).

###  Inclusion and exclusion criteria

 All glaucoma patients ≥ 40 years who were fully informed about study variables were included in the study, whereas the patients with insufficient information were excluded from the study.

###  Statistical analysis

 The endpoints can be strongly correlated in this study, implying an appropriate statistical model that expresses the dependence between the times of two events. However, such standard tools as Cox regression, are not suitable for the simultaneous analysis of the two event times. This study provides an advanced statistical model that incorporates the dependence between the two endpoints in terms of copulas.^24-26^ Therefore, in this particular study, the copula model was applied for the bivariate survival analysis. A copula can be used to link two event times by specifying their dependence structure. Copulas have primary and direct applications in the simulation of dependent variables. The copula function is used to model the bivariate survival data.^27^

 Let *T*_1_*,T*_2_ be the two bivariate event times, with the marginal survival function $S_{j}\left(t_{j}\right)=P_{r}\left(T_{j}>t_{j}\right),j=1,2$ and the joint survival function $S\left(t_{1},t_{2}\right)=P_{r}\left(T_{1}>t_{1},T_{2}>t_{2}\right)$ , assuming that there are *n* independent subjects in a study. Where *T*_1_*,T*_2_ are subject to the right censoring for the subject *i = 1,2,…,n*, we observe $D_{i}=\left\{\left(Y_{ij},\sigma_{ij},Z_{ij}\right);Y_{ij}=\min\left(T_{ij},c_{ij}\right),\sigma_{ij}=I\left(T_{ij}<c_{ij}\right),j=1,2\right\}$ , where *c*_ij_ is the censoring time, *T*_ij_*σ*_ij_ are the censoring indicator, and* Z*_ij_ is the vector of covariates. By Sklar’s theorem,^28^ there exists a unique function *c*_η_ that connects two marginal survival functions to the joint survival function so long as the marginal survival function *S*_j_ is continuous.


$$S\left(t_{1},t_{2}\right)=c_{\eta }S_{1}\left(t_{1}\right),S_{2}\left(t_{2}\right),t_{1},t_{2}>0$$

Here, the function *c*_η_ is called copula, and its parameter *η* measures the dependence between the two margins. It allows the dependence to be modeled separately from the marginal distribution. A commonly used family of copula functions is the Archimedean copulas which include Clayton copula, Gumbel-Hougaard copula, Frank copula, and Joe copula.^29,30^

###  Model selection, diagnostics, and goodness of fit test

 In this study, the Akaike information criterion (AIC) was used to compare different candidate models, and the model with a relatively small value of AIC has been considered to be a better fit.^31,32^ Model diagnostics is one of the fundamental steps to confirm the model assumptions after obtaining a final model using the model selection method. In this study, a diagnostic test was applied for bivariate survival data with families of copula model which is based on Kendall’s τ, Q-Q plot2, and Scatter plot to verify the appropriateness of the distributional assumptions and the adequacy of the model assumed.^27^ Overall, the goodness-of-fit test for the fitted models was assessed using the Pseudo Maximum Likelihood Estimator.^33^

## Results

###  Baseline information and descriptive statistics

 In total, 211 (66.9%) out of 315 glaucoma patients were blind, whereas 104 (33.1%) patients were censored. Glaucoma patients were in the age range of 40-84 years with a median survival time of 12 months. The minimum and maximum follow-up times in this study were determined to be one month and 60 months, respectively. During the study period, 107 (33.9%) and 208 (66.1%) patients were female and male, respectively, about 190 (60.3%) and 125 (39.7%) of whom were living in rural and urban areas. In total, 174 (55.2%) and 111 (35.3%) patients had abnormal IOP and were diabetic, respectively. Most of the patients were treated with Timoglue medication, and 88 (27.9%) out of 128 (40.6%) patients were blind. The remaining 97 (30.8%) and 90 (28.6%) patients were treated with Diamox and timolol medication, respectively (Table 1). Moreover, 97 (30.7%) and 98 (31.1%) patients had a medium and long treatment duration, and the other 120 (38.2%) patients had a short treatment duration. A total of 36 (11.4%), 66 (21%), 145 (46.0%), and 68 (21.6%) patients had early, moderate, advanced, and absolute glaucoma, respectively. Similarly, 141 (44.8%) patients had a normal or ≤ 0.7 cup-disk ratios, and the remaining 174 (55.2%) patients did not have a normal or greater than 0.7 cup-disk ratio. In total, 65 (20.6%) female and 146 (46.3%) male patients were blind, respectively. Similarly, 132 (41.9%) and 79 (25.0%) blind patients were from rural and urban areas, respectively. Eventually, 77 (24.4%), 60 (19.0%), and 74 (23.5%) patients were blind and had short, medium, and long treatment duration, respectively (Table 1).

**Table 1:** Baseline characteristics of categorical variables of glaucoma patients

**Variables **	**Patients**		**Blind**		**Non-blind**		**Median (m)**
	**No.**	**%**	**No.**	**%**	**No.**	**%**	
Gender							
Female	107	33.9	65	20.6	42	13.4	12
Male	208	66.1	146	46.3	62	19.7	12
Place of residence							
Rural	190	60.3	132	41.9	58	18.5	12
Urban	125	39.7	79	25	46	14.6	12
Diabetic disease							
No	204	64.7	128	40.6	76	24.2	12
Yes	111	35.3	83	26.3	28	8.9	12
Intraocular Pressure							
Normal	141	44.8	74	23.4	67	21.3	12
Not normal	174	55.2	137	43.5	37	11.8	12
Type of medication							
Timoglue	128	40.6	88	27.9	40	12.7	12
Diamox	97	30.8	59	18.7	38	12.1	12
Timolol	90	28.6	64	20.3	26	8.3	12
Duration of treatment							
Short	120	38.2	77	24.4	43	13.7	10
Medium	97	30.7	60	19.0	37	11.8	12
Long	98	31.1	74	23.5	24	7.6	24
Cup-disk ratio							
≤ 07	118	37.5	58	18.4	60	19.1	12
> 0.7	197	62.5	153	48.5	44	14.0	12
Stage of glaucoma							
Early	36	11.4	10(	3.1	26	8.3	14
Moderate	66	21	18	5.7	48	15.3	20
Advanced	145	46.	115	36.5	30	9.5	12
Absolute	68	21.6	68	21.6	0	0.0	10

Source: Jimma University Medical Center, Ethiopia; from January 1, 2016, to August 30, 2020.

###  Multivariable analysis

 Univariable analysis of all parameters regarding glaucoma patients’ time to blindness was analyzed before choosing variables for the model. The multivariable semi-parametric model included the variables that were significant at a cut of point 0.25 in univariable analysis. Accordingly, at a 0.05 level of significance, gender, place of residence, diabetic disease, IOP, type of medication, duration of treatment, cup-disk ratio, and the stage of glaucoma were significant and affected the time it took for glaucoma sufferers to go blind (Table 2).

**Table 2:** Multivariable analysis of glaucoma patient’s data at Jimma University Medical Center, 2016-2020 (n = 315)

**Covariates**	**β**	**HR**	***P*** ** value**
Gender			
Female	Ref.		
Male	0.005	1.005	0.010
Place of residence			
Rural	Ref.		
Urban	-0.348	0.707	0.002
Diabetic disease			
No	Ref.		
Yes	0.157	1.169	0.009
Intraocular Pressure			
Normal	Ref.		
Not normal	0.091	1.095	0.003
Type of medication			
Diamox	Ref.		
Timoglue	-0.121	0.887	0.003
Timolol	-0.024	0.976	0.008
Duration of treatment			
Long	Ref.		
Medium	-1.125	0.325	0.001
Short	-1.422	0.242	0.001
Cup-disk ratio			
> 0.7	Ref.		
≤ 0.7	-0.235	0.791	0.011
Stage of glaucoma			
Absolute	Ref.		
Early	-0.542	0.582	0.002
Moderate	-0.725	0.485	0.001
Advanced	-0.121	0.887	0.003

Source: Jimma University Medical Center, Ethiopia; from January 1, 2016, to August 30, 2020.

 Based on the results of the semi-parametric model, the level of significance was determined at 0.05. The effects of each predictor on the response variable were determined considering the Exp (β), which is the hazard ratio and can be interpreted as the predicted change in the hazard for a unit increase in the predictor. After controlling other variables as constant, the estimated hazard ratio of the blindness of male to female patients was HR = 1.005. The result indicates that the blindness risk of male patients was 1.005 times higher than that in female patients (*P* = 0.01). Similarly, the estimated hazard ratios of blindness for patients who took Timoglue and timolol medication to those who took Diamox medication were HR = 0.887 and HR = 0.976, respectively, indicating that the blindness risk of the patients who took Timoglue and timolol medication was estimated to be 0.887 (*P* = 0.003) and 0.976 (*P* = 0.008) times less than that in patients who took Diamox medication, respectively. Eventually, the estimated hazard ratios of blindness for patients who had early, moderate, and advanced glaucoma to patients who had absolute glaucoma were obtained at HR = 0.582, HR = 0.485, and HR = 0.887, respectively. This indicated that the blindness risk in patients who had early, moderate, and advanced glaucoma was 0.582 (*P* = 0.002), 0.485 (*P* = 0.001), and 0.887 (*P* = 0.003) times less than that in patients who had absolute glaucoma, respectively (Table 2).

###  Parametric model selection and comparison 

 In this particular study, we considered the Archimedean copula families with a marginal distribution, such as Weibull, Gompertz, and log-logistic. The AIC values of Clayton, Gumbel, Frank, and Joe copula families with Weibull margin were 982.214, 1042.566, 3627.856, and 1090.264, respectively. The Kendall’s tau value of Clayton with Weibull margin was 0.83, this indicates there is high dependence between the time to blindness of left and right eyes of glaucoma patients. Accordingly, the Clayton copula model with Weibull distribution was the minimum among other AIC values of the Archimedean copula models, indicating it to be the best fit for the glaucoma patients’ data sets.

###  A parametric model for various copula family

 The Clayton copula model with Weibull margin of the predictor variables, such as place of residence, age, gender, type of medication, diabetic disease, stage of glaucoma, IOP, duration of treatment, and the cup-disk ratio, was significantly associated with the time of the blindness of the left and right eyes of glaucoma patients at 0.05 level of significance (Table 3). From this analysis, the estimated hazard ratio of blindness for patients’ age was exp (0.01) = 1.01, implying that for a unit increased in age, the hazard ratio of the blindness of patients was significantly increased by 1.01 (*P* = 0.002), keeping all other variables constant. The estimated hazard ratio of the blindness in patients who lived in urban areas to patients who lived in the rural areas was exp (-0.23) = 0.79, indicating that the blindness risk in patients who lived in urban areas was 0.79 times (*P* = 0.002) less than that in patients who lived in rural areas (Table 3).

**Table 3:** Parametric model for Clayton copula with Weibull margin for glaucoma patients’ data set

**Variables**	**Estimate**	***P*** ** value**
Lambda	0.28	0.002
K	1.65	0.002
Age (y)	0.01	0.002
Place of residence		
Rural	Ref.	
Urban	-0.23	0.002
Gender		
Female	Ref.	
Male	0.02	0.002
Type of medication		
Diamox	Ref.	
Timoglue	-0.06	0.002
Timolol	-0.04	0.002
Diabetic disease		
No	Ref.	
Yes	0.04	0.002
Stage of glaucoma		
Absolute	Ref.	
Early	-0.33	0.002
Moderate	-0.51	0.002
Advanced	-0.04	0.002
Intraocular Pressure		
Normal	Ref.	
Not normal	0.03	0.002
Duration of treatment		
Short	-2.48	0.002
Medium	-1.26	0.002
Long	Ref.	
Cup-disk ratio		
> 0.7	Ref.	
≤ 07	-0.02	0.002
Eta	9.57	1.000

K: The scale parameters of the baseline Weibull distribution Source: Jimma University Medical Center, Ethiopia; from January 1, 2016, to August 30, 2020.

 Similarly, the estimated hazard ratio of blindness in male to female patients was exp (0.02) = 1.02. Based on the results, the blindness risk of male patients was 1.02 (P = 0.002) times higher than that in female patients. The estimated hazard ratios of blindness for patients who took Timoglue and timolol medication to patients who took Diamox medication were exp (-0.06) = 0.94 and exp (-0.04) = 0.96, respectively, indicating that the risk of the blindness in patients who took Timoglue and timolol medication was estimated to be 0.94 (*P* = 0.002) and 0.96 (*P* = 0.002) times less than that in patients who took Diamox medication, respectively (Table 3).

 The results indicated that the estimated hazard ratio of blindness in patients who had diabetic disease relative to patients who had no diabetic disease was exp (0.04) = 1.04, indicating that the blindness risk in patients who had the diabetic disease was 1.04 (*P* = 0.002) times higher than that in patients who had no diabetic disease. The estimated hazard ratio of blindness for patients who had early, moderate, and advanced glaucoma relative to patients who had absolute glaucoma were exp (-0.33) = 0.72, exp (-0.51) = 0.60, and exp (-0.04) = 0.96, respectively, indicating that blindness risk in patients who had early, moderate, and advanced glaucoma were 0.72 (*P* = 0.002),0.60 (*P* = 0.002), and 0.96 (*P* = 0.002) times less than the blindness risk in patients who had absolute glaucoma, respectively (Table 3).

**Figure 1:**
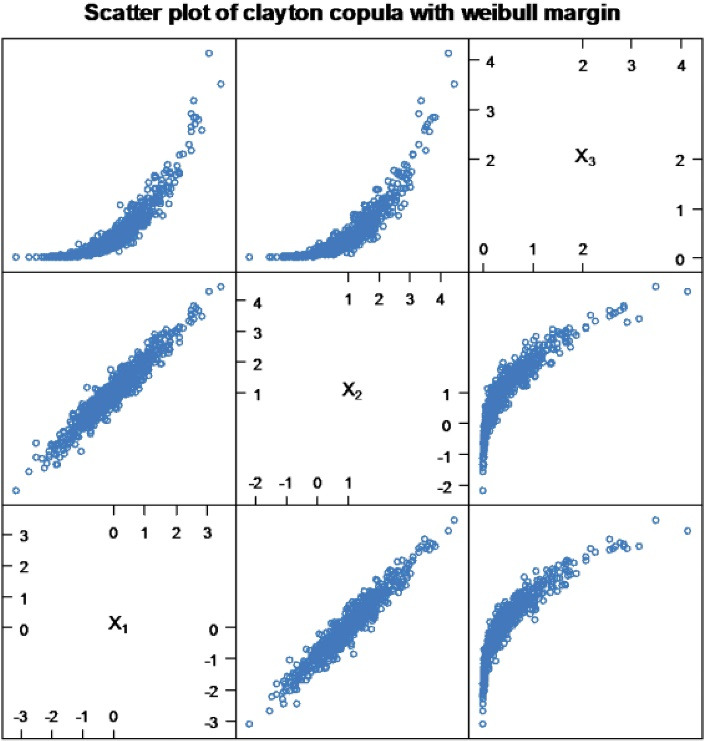
Scatter plot: testing the degree of dependence of Clayton copula with Weibull margin, based on Kendall’s tau value.

 In line with the above interpretation, the estimated hazard ratio of blindness in patients who had abnormal IOP to patients who had normal IOP was exp (0.03) = 1.03, indicating that the blindness risk in patients who had abnormal IOP were 1.03 (*P* = 0.002) times higher than the blindness risk in patients who had normal IOP. The estimated hazard ratios of blindness in patients who had a short and medium duration of treatment to patients who had a long treatment duration were exp (-2.48) = 0.084 and exp (-1.26) = 0.28, respectively, indicating that the blindness risk in patients who had a short and medium treatment duration was 0.084 (*P* = 0.002) and 0.28 (*P* = 0.002) times less than that in patients who had a long treatment duration, respectively. Eventually, the estimated hazard ratio of blindness in patients who had a cup-disk ratio ≤ 0.7 to patients who had a cup-disk ratio > 0.7 was exp (-0.02) = 0.98, indicating that blindness risk in patients who had a cup-disk ratio ≤ 0.7 was 0.98 (*P* = 0.002) times less than that in patients who had cup-disk ratio > 0.7 (Table 3). Therefore, a high dependence existed between the time of the blindness of the left and right eyes in glaucoma patients (Figure 1).

###  Model diagnostics

 Once the models are fitted, all the necessary model assumptions should be verified. Standard types of plots are often applied to check and validate the assumptions behind the copula model.

**Figure 2:**
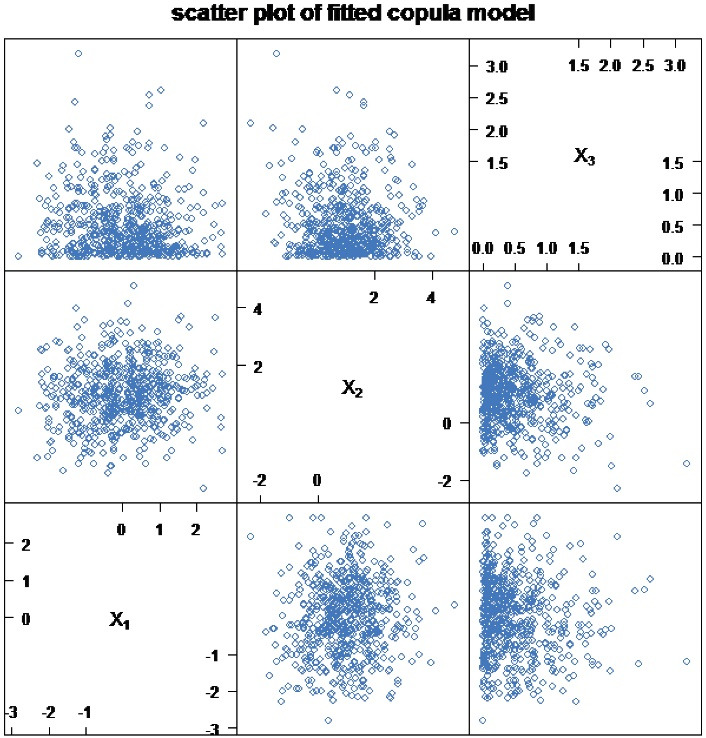
Scatter plot of fitted Clayton copula model.

 The overall goodness-of-fit test of the copula model can be assessed using the scatter plot. Figure 2 shows the lack of a systematic pattern, indicating that the model fits the data.

## Discussion

 A bivariate survival model such as copula model were used to estimate the dependence between the two responses (left and right eyes) of glaucoma patients, by considering the explanatory variables. The copula framework allows the dependency structure between the responses to be isolated from their marginal distributions.^25^The method consists of introducing copulas as an alternative to the correlation coefficient commonly used as a measure of dependence. An algorithm based on the marginal distributions of random variables is applied to construct the Archimedean copulas. An alternative dependence measure is a copula that overcomes the limitations of correlation as a measure of dependence.^25,26,34^ A copula has proved to be useful in a variety of modeling situations and is a relatively new concept that has been applied in survival data analysis.^35,36^

 Copulas are functions that join or couple multivariate distribution functions to their one-dimensional marginal distribution function. Advantages of using copulas in modeling include (i) allowance to model both linear and non-linear dependence, (ii) arbitrary choice of a marginal distribution, and (iii) capability of modeling extreme endpoints. This study aimed to describe the bivariate survival models, such as the copula model, which can be employed as an alternative for any multivariate data set and estimates the dependence between correlated endpoints. Implementation of Archimedean copula models, based on the copula approach, has been illustrated through the analysis of glaucoma patients’ data.^28^

 In this study, Archimedean copula family models, such as Clayton, Gumbel, Frank, and Joe were explored for modeling time to the blindness of the left and right eyes in the glaucoma patients. The assumption of the model was checked using the measure of dependence Kendall’s tau, normal Q-Q plot2, and scatter plot. All plots of the original data indicated that there was not any deviation from the model assumption and no need for transformation. The analyses of the bivariate survival data, such as parametric bivariate survival analysis, were used to investigate the determinant factors of the time to the blindness of the left and right eyes. The baseline parameter of the Clayton copula model with Weibull margin was statistically significant in the model, indicating the correlation between the bivariate responses. This finding was consistent with those obtained by Sun et al.^37^Based on the results, the statistical significance of the estimated parameter is a piece of evidence indicating the Clayton copula model to be a better fit than other models.

 The prognostic factors considered in this study included age, gender, place of residence, type of medication, diabetes disease, stage of glaucoma, treatment duration, IOP, and cup-disk ratio. All the above predictor variables were found to be the determinant factors for the time to the blindness of the left and right eyes in glaucoma patients, using the univariable analysis. Therefore, these covariates were used in the multivariable analysis to compare the parametric Archimedean copula models. Analysis using the best model (i.e. the Clayton copula model) with the Weibull margin showed that age was an important socio-demographic factor for the time to the blindness in glaucoma patients, implying that the blindness risk increases with age. Similarly, the results of another study conducted by Rossetti et al^38^ showed that higher age was a significant risk factor for blindness in glaucoma patients. Moreover, time to the blindness of glaucoma patients was significantly associated with the type of medication and duration of treatment. Moreover, the risk of blindness was reduced in the glaucoma patients who took Timoglue and timolol medication and those who had a short and medium treatment duration. Consistently, the results of another study^39^ showed that the use of various class of glaucoma medication and short and medium treatment duration reduced the hazard of death or blindness.

 In this study, the risk of blindness in glaucoma patients was associated with IOP and diabetes. The risk of blindness in glaucoma patients who had abnormal IOP or IOP greater than 21 mmHg was higher compared to patients who had normal IOP, and the patients with diabetes had a high risk of blindness. However, these variables were insignificant in the study conducted by de Voogd et al,^40^ indicating that the results obtained in this study were more reliable than those obtained by de Voogd et al. In addition, the predictor variable of the stage of glaucoma was significantly associated with the time to the blindness of glaucoma patients in this study. The glaucoma patients who had early, moderate, and advanced glaucoma had a lower risk of blindness, compared to those who had absolute glaucoma. This result contradicts the findings of Caprioli & Coleman^41^and Drance et al.^42^ Regarding the cup-disk ratio, the risk of blindness in patients who had a cup-disk ratio greater than 0.7 was higher compared to patients who had a cup-disk ratio less than or equal to 0.7. This finding was consistent with those obtained by Gardiner et al^31^ which showed an increased incidence of blindness in patients with a cup-disc ratio larger than 0.7.

 The dependence of Kendall’s tau value of Clayton copula model with Weibull margin was estimated to be τ = 0.83, showing an extreme dependence with time to the blindness of the left and right eyes in glaucoma patients. The study results were in line with those obtained by Kendall.^43^ Eventually, the results obtained in this study were confirmed by Sun et al,^37^ whose results showed the significance of the copula model for the bivariate data set. Therefore, it can be concluded that the Clayton copula model is the best fit for the data compared to other Archimedean copula family models.

###  Strengths and limitations of the study

 The application of an advanced statistical model that simultaneously incorporates time to two events (i.e. time to the blindness of the left and right eyes in glaucoma patients) is a strength of this study. Regarding the limitations of the present study, one can refer to the fact that this study has been conducted based on secondary data which might be incomplete and biased. Moreover, there was poor data recording on different charts of patients ’information. As for the Archimedean copula family models, some outputs were missing due to the lack of computer computing capacity and the unavailability of enough software packages for the copula model.

## Conclusion

 Overall, regarding the performance of Archimedean copula family models in terms of model parsimony and goodness of fit test, the Clayton copula model with Weibull margin was a better fit based on its AIC value. Place of residence, age, gender, type of medication, diabetes, stage of glaucoma, duration of treatment, IOP, and cup-disk ratio were significantly associated with and affected by the time to the blindness of the left and right eyes in glaucoma patients. Awareness should be given to the community to reduce the burden of glaucoma.

**Highlights**

- The median time to the blindness of left and right eyes was determined to be 12 months.
- The copula models were applied to study the dependence between the time of the blindness of the left and right eyes in glaucoma patients.
- There was a high dependence between the time of the blindness of the left and right eyes in glaucoma patients.
- Clayton copula model with Weibull margin had the minimum AIC value and was the best model among other Archimedean copula family models.

## Acknowledgments

 The authors would like to acknowledge Jimma University, the College of Natural Sciences, and the Research and Postgraduate Coordinating Office for their financial support and the permission to carry out this study.

## Authors’ contribution

 MGG contributed to the study concept, designing, and drafting of the manuscript. RHB, DKD, and KSW KTD participated in data collection and drafted the manuscript, and GMA reviewed the final manuscript.

## Availability of data

 The data sets analyzed in this study are available from the corresponding author on a reasonable request.

## Conflict of interest

 The authors declare that they have no competing interests regarding the publication of the present study.

## Funding

 This study was financially supported by the College of Natural Sciences, Jimma University, Jimma, Ethiopia. The supporting bodies had no role in data collection, analysis, and preparation of the manuscript or in the decision to publish.
